# A cell‐centered finite volume formulation of geometrically exact Simo–Reissner beams with arbitrary initial curvatures

**DOI:** 10.1002/nme.6994

**Published:** 2022-05-12

**Authors:** Seevani Bali, Željko Tuković, Philip Cardiff, Alojz Ivanković, Vikram Pakrashi

**Affiliations:** ^1^ School of Mechanical and Materials Engineering University College Dublin Dublin 4 Ireland; ^2^ SFI MaREI Centre University College Dublin Dublin 4 Ireland; ^3^ Bekaert University Technology Centre, School of Mechanical and Materials Engineering University College Dublin Dublin 4 Ireland; ^4^ Dynamical Systems and Risk Laboratory, School of Mechanical and Materials Engineering University College Dublin Dublin 4 Ireland; ^5^ UCD Centre for Mechanics University College Dublin Dublin 4 Ireland; ^6^ Faculty of Mechanical Engineering and Naval Architecture University of Zagreb Zagreb Croatia; ^7^ SFI I‐Form Centre University College Dublin Dublin 4 Ireland; ^8^ UCD Centre of Adhesion and Adhesives University College Dublin Dublin 4 Ireland

**Keywords:** block‐coupled, finite volume method, geometrically exact beam, Newton–Raphson, total Lagrangian

## Abstract

This article presents a novel total Lagrangian cell‐centered finite volume formulation of geometrically exact beams with arbitrary initial curvatures undergoing large displacements and finite rotations. The choice of rotation parameterization, the mathematical formulation of the beam kinematics, conjugate strain measures, and the linearization of the strong form of governing equations are described. The finite volume based discretization of the computational domain and the governing equations for each computational volume are presented. The discretized integral form of the equilibrium equations is solved using a block‐coupled Newton–Raphson solution procedure. The efficacy of the proposed methodology is presented by comparing the simulated numerical results with classic benchmark test cases available in the literature. The objectivity of strain measures for the current formulation and mesh convergence studies for both initially straight and curved beam configurations are also discussed.

## 1 INTRODUCTION

The mathematical modeling of nonlinear beams has flourished in the past few decades with their applicability spanning various fields of engineering. A concise review of several beam formulations, the consistent derivation of 1D beam theories from the general 3D continuum mechanics and a systematic nomenclature of the existing beam models are presented in Meier et al..^1^ These formulations, in particular, and the numerical computations of solid mechanics problems, in general, are mostly analyzed using the finite element (FE) approach; however, since the 1980s, the applicability of the simple and conservative finite volume (FV) methods to solid mechanics problems has become increasingly popular and is evolving rapidly.^2^ Particularly in the context of beams, Fallah et al.^3^, ^4^, ^5^ have presented FV implementations of Euler–Bernoulli and shear‐deformable Timoshenko beam theories for both straight and curved beam configurations. Furthermore, several authors have presented the stability^6^, ^7^, ^8^ and vibration^9^, ^10^ analysis of beams/columns. The analysis of thin and thick plates using different variants of FV methods like cell‐centered FV,^11^, ^12^, ^13^ cell‐vertex FV^14^ and the orthogonal meshless variant of FV^15^ and their comparison with traditional FE formulations has also been explored in the literature. A common conclusion from the aforementioned references is that the FV formulation of beams and plates does not suffer from the “shear‐locking” phenomenon for both thin Timoshenko beams and thin Mindlin–Reissner plate analysis, unlike traditional fully integrated FE methods that require special techniques like reduced/selective integration and mixed interpolation methods to avoid locking effects. A shear locking study to demonstrate the effectiveness of the FV formulation in the context of geometrically exact beam theory is presented towards the end of Section 4.2.

The geometrically exact Simo–Reissner beam formulation is the most general nonlinear 3D beam theory capable of dealing with finite displacements and rotations. The FV formulation of geometrically exact beams was first investigated by Tukovic et al.^16^ The current work builds on the formerly developed formulations for quasi‐static shear‐deformable geometrically exact beams and provides a comparison between the FE based classic benchmark cases and the proposed FV methodology. The primary motivation of extending the Simo–Reissner formulation to an FV discretization is to bring solid and fluid numerical formulations under one unified approach allowing multi‐physics problems like fluid‐solid interaction to be addressed using a single numerical technique. For instance, a direct application of this FV formulation is the simulation of slender structures like cables and mooring lines and their interaction with oceanic waves. An additional interesting feature of FV methods is the strong local (and global) conservation of governing equations. Typically, in FV, the spatial domain is discretized into non‐overlapping cells/control volumes, which ensures an exact balance of forces across cell boundaries at a local level, and global conservation is automatically achieved. This is in contrast to the FE techniques that have locally overlapping integration domains and hence, at the local element level, conservation of governing laws is ensured only in an average sense. A critical and comprehensive comparison of FV and FE techniques in the field of solid mechanics can be found in the review article of Cardiff and Demirdžić.^2^ To the best of the authors' knowledge, this is the first article to present an FV formulation for beams subjected to finite displacements and rotations.

The formulations of a beam in a geometrically exact sense considering 3D rotations and its FE approximation were first proposed by Simo et al..^17^, ^18^ The involvement of finite rotations, elements of the nonlinear differentiable manifold SO(3) and the complexity of interpolating large rotations^19^, ^20^ led to three major techniques of addressing them, namely, (a) incremental and total rotational vector based parameterizations,^21^, ^22^, ^23^, ^24^ (b) quaternion based rotation interpolation,^25^, ^26^, ^27^, ^28^ and (c) co‐rotational beam formulations,^29^, ^30^ to name a few. Romero^31^ presents a concise review of different rotation interpolations used for geometrically exact beams. In the current work, the finite rotations are parameterized using rotational vectors and the rotational strain measures are updated using incremental rotation vectors from the previously converged configuration of the beam. The loss of objectivity in strain measures for FE approximation of interpolated rotations was first pointed out by Crisfield and Jelenić^32^ which was followed by strain invariant formulations of the geometrically exact beams.^33^, ^34^, ^35^, ^36^ For the current work, the objectivity of adopted strain measures are numerically verified using a test case presented in Section 4.1. As an alternative to rotation/quaternion based interpolation, director based formulations catered for continuum‐based beam elements are presented in Eugster et al.^37^ Extension of 1D linear elastic constitutive models used by Simo et al.^17^, ^18^ to finite 3D elasticity using general 3D kinematic measures like deformation gradients can also be found in the literature.^38^, ^39^ In a recent contribution, Meier et al.^40^ developed a geometrically exact Kirchhoff–Love formulation for slender rod geometries. For a general review of other research advances in this field, like discrete Cosserat rod kinematics, implementation of different time‐stepping schemes in beam multi‐body dynamics, the inclusion of nonlinear constitutive laws, and enhanced kinematics of beams, the interested readers are referred to articles by Meier et al.^1^ and Chadha and Todd.^41^

This article is structured as follows: Section 2 outlines the mathematical model in total Lagrangian form. The FV discretization of the mathematical model is described in Section 3. In Section 4, the proposed methodology is evaluated on five complementary benchmark test cases, where predictions are compared to analytical solutions and existing FE benchmarks.

## 2 MATHEMATICAL MODEL

In this section, the mathematical formulation for a 3D quasi‐static, shear‐deformable geometrically exact Simo–Reissner beam is summarized. Subsequently, the governing equilibrium equations of spatial forces and moments, the constitutive relations and their linearized formulation are described.

### 2.1 Kinematic description

**FIGURE 1:**
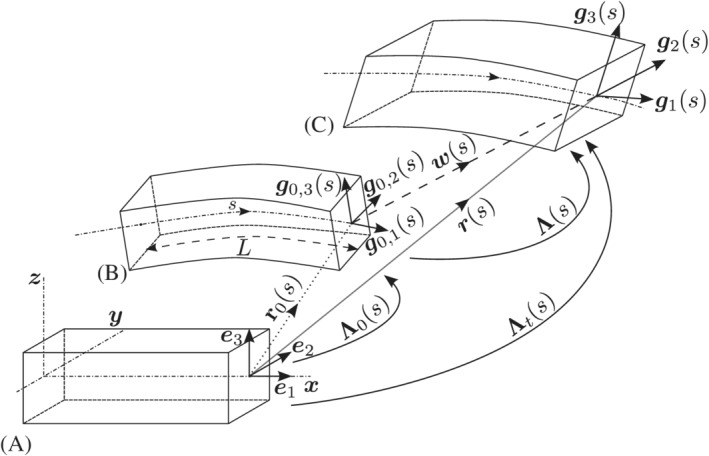
Beam kinematics: (A) Reference, (B) initial, and (C) deformed configurations of the beam, respectively

A total Lagrangian formulation is adopted to describe the large deformations of the beam model. Accordingly, a right‐handed fixed, reference Cartesian frame defined by the orthonormal basis vectors $e_{1}={\left[1,0,0\right]}^{T}$, $e_{2}={\left[0,1,0\right]}^{T}$, and $e_{3}={\left[0,0,1\right]}^{T}$ is specified (Figure 1). In the reference (i.e., material) configuration, the mean line of a beam (i.e., line of centroids of the beam cross‐sections) is straight and parallel to the basis vector $e_{1}$ and the cross‐sections of the beam are orthogonal to the basis vector $e_{1}$. The principal axis of inertia of the beam cross‐sections are directed along the basis vectors $e_{2}$ and $e_{3}$.

An initial (stress‐free) configuration of the beam mean line is defined by the space curve $r_{0}(s)$, where s∈[0,L] is the beam‐length and L is the initial length of the beam (Figure 1). To describe the continuous kinematic description of the moving mean line curve of the beam, a body‐attached right handed orthonormal base vectors $g_{0,1}(s)$, $g_{0,2}(s)$, and $g_{0,3}(s)$ are defined by the Frenet–Serret formulas, where the base vector $g_{0,1}(s)$ is directed along the initial mean line so that

(1) $$g_{0,1}(s)=r_{0}^{\prime }(s)$$

and the base vectors $g_{0,2}(s)$ and $g_{0,3}(s)$ are directed along the principal axis of inertia of the cross‐section at s. The prime ${\left(\cdot \right)}^{\prime }$ operator in Equation (1) and hence forward wherever used, denotes a derivative with respect to arc‐length parameter s, that is, ${\left(\cdot \right)}^{\prime }\equiv \frac{\partial (\cdot )}{\partial s}$. For an initially straight beam, the base vectors $g_{0,i}$ and the reference bases $e_{i}$ coincide, and the $g_{0,1}(s)\equiv e_{1}(s)$.

The orthonormal basis $g_{0,1}(s)$, $g_{0,2}(s)$, $g_{0,3}(s)$ of the local frame and the orthonormal bases $e_{1}$, $e_{2}$, $e_{3}$ of the global Cartesian frame are related through a linear transformation $\Lambda_{0}(s)$ as

(2) $$g_{0,i}(s)=\Lambda_{0}(s)e_{i},\;i=1,2,3,$$

where $\Lambda_{0}(s)\in \text{SO}(3)$ is the initial two‐point second‐order orthogonal rotational tensor field that defines the orientations of beam cross‐sections with respect to the reference basis. Hence, the initial configuration of the beam is fully defined by the position vector $r_{0}(s)$ of the beam mean line and orientation of the cross‐section at s via the orthogonal rotation tensor $\Lambda_{0}(s)$.

The deformed configuration of the beam mean line is defined by a space curve r(s) and the orientation of the cross‐sections via the moving spatial bases $g_{1}(s)$, $g_{2}(s)$, $g_{3}(s)$. Contrary to the initial configuration, the basis vector $g_{1}(s)$ need not be directed along the deformed mean line since the beam model is capable of representing shear deformations. The base vectors $g_{2}(s)$ and $g_{3}(s)$, however, are still directed along the principal axes of the cross‐section at s and the orthonormal bases $g_{1}(s)$, $g_{2}(s)$, $g_{3}(s)$ are related to $g_{0,1}(s)$, $g_{0,2}(s)$, $g_{0,3}(s)$ bases by the linear transformation

(3) $$g_{i}(s)=\Lambda (s)g_{0,i}(s)=\Lambda (s)\Lambda_{0}(s)e_{i},\;i=1,2,3,$$

where Λ(s)∈SO(3) is the relative rotational matrix which rotates beam cross‐sections from the initial to the deformed configuration. Additionally, the deformed mean line of the beam can also be defined using the mean line displacement vector w(s) and the mean position vector of the initial configuration by,

(4) $$r(s)=r_{0}(s)+w(s).$$

The position vector of the deformed mean line r(s) and the orientation of the $g_{i}(s)$ triad given by Λ(s) attached to the cross‐section at s, (r(s),Λ(s)) fully define the deformed configuration of the beam. Orientation of the beam cross‐sections in the deformed configuration can also be defined with respect to the fixed reference bases by the total rotation matrix,

(5) $$\Lambda_{t}(s)=\Lambda (s)\Lambda_{0}(s).$$

### 2.2 Balance equations and strain measures

Assuming that the deformation from the initial to the deformed configuration is caused by distributed external forces and torques f and t per unit of reference arc length, respectively, the strong differential form of the balance equations are given as,^17^

(6) $$n^{\prime }+f=0,$$

(7) $$m^{\prime }+r^{\prime }\times n+t=0,$$

where n and m are the vectors of spatial internal forces and moments acting over the cross‐section at s and “×” symbol denotes the cross product between two vectors. The corresponding material counterparts (N and M) are related to n and m via the pull‐back mapping $\Lambda_{t}^{T}$ as $N=\Lambda_{t}^{T}n$ and $M=\Lambda_{t}^{T}m$.^17^

The equivalent strong integral form of the above balance equations over a length, L can be expressed as,

(8) $$\int_{L}n^{\prime }dL+\int_{L}fdL=0,$$

(9) $$\int_{L}m^{\prime }dL+\int_{L}(r^{\prime }\times n)dL+\int_{L}tdL=0.$$

The required strain measures follow from a geometrically exact beam theory, where the relationships between the beam configuration and the strain measures are consistent with the virtual work principle and the equilibrium equations at a deformed state regardless of the magnitude of displacements, rotations, and strains.^18^ To that end, the equivalent strain‐configuration relationships involve three translational strains and a skew‐symmetric tensor of the three rotational strains^17^, ^22^ (from here on, the argument (s) is dropped from the terms for clarity):

(10) $$\Gamma =\Lambda_{t}^{T}r^{\prime }-\Lambda_{t}^{T}g_{1},$$

(11) $$\hat{K}=\Lambda_{t}^{T}\Lambda_{t}^{\prime }-\Lambda_{0}^{T}\Lambda_{0}^{\prime },$$

where Γ and K are (material) translational and rotational strain measures, respectively. The hat $\hat{\left(\cdot \right)}$ operator denotes a skew‐symmetric matrix associated with the corresponding (pseudo)‐vector given by the relation, $a\times h=\hat{a}h$, ∀ $h\in {\mathbb{R}}^{3}$. Physically, the strain vector Γ represents axial tension (first entry) and shear deformation whereas the (material) rotational strain vector K represents torsion (first entry) and bending deformation of the beam body. These strain measures are the energy‐conjugate pairs to the stress resultants (N and M).

In this work, the relative rotation matrix Λ is parameterized in terms of its rotational vector ψ as

(12) $$\Lambda (\psi )=\exp(\hat{\psi })=I+\frac{\sin\psi }{\psi }\hat{\psi }+\frac{1-\cos\psi }{\psi^{2}}\hat{\psi }\hat{\psi },$$

where I is a 3×3 identity matrix and ψ is the magnitude of rotation vector ψ. The $\hat{\psi }\in so(3)$ is the skew‐symmetric tensor living in the tangent space of SO(3) at Λ and its exponentiation yields the finite rotation Λ∈SO(3). For a rotation vector based parameterization, an alternative expression for evaluating incremental (material) rotational strain vector ΔK, as demonstrated in References 20, 22, and 33 is given by,

(13) $$\Delta K=\Lambda_{t}^{T}T^{T}(\Delta \psi )\Delta \psi^{\prime },$$

where the tangent operator T(ψ)∈so(3) is defined as (“⊗” denotes the dyadic product between two vectors),

(14) $$T(\psi )=\frac{\sin\psi }{\psi }I+\frac{1}{\psi^{2}}\left(1-\frac{\sin\psi }{\psi }\right)\psi ⊗\psi +\frac{1-\cos\psi }{\psi^{2}}\hat{\psi }.$$

### 2.3 Constitutive relations

The present study is limited to linear hyperelastic materials whose length‐specific stored energy function is given by,

(15) $${\tilde{\Pi }}_{\text{int}}=\frac{1}{2}\Gamma^{T}C_{N}\Gamma +\frac{1}{2}K^{T}C_{M}K.$$

For this hyperelastic model, the material internal forces N and moments M are linearly related to the material strain measures (Γ and K) as, $N=C_{N}\Gamma$ and $M=C_{M}K$ where $C_{N}=\text{diag}[EA,GA_{2},GA_{3}]$ and $C_{M}=\text{diag}[GJ,EI_{2},EI_{3}]$ are constant diagonal constitutive matrices. Here, E and G denote the elastic and shear moduli of the material, respectively, and $A_{p}$ and $I_{p}$ (p=1,2,3), are the effective (material) areas and area moments about the principal axes of inertia, respectively ($J\equiv I_{1}$).

### 2.4 Linearization of balance equations

The nonlinear exact spatial forces and moments^17^ given in Equations (16) and (17) need to be linearized about a certain base equilibrium point to apply the iterative Newton–Raphson scheme and successively refine their values toward convergence.

(16) $$n=\Lambda_{t}C_{N}\Gamma ,$$

(17) $$m=\Lambda_{t}C_{M}K.$$

Accordingly, the spatial forces n and spatial moments m are linearized about their corresponding explicit values calculated using the values of strains and curvatures obtained from the previous iteration as follows,

(18) $$L[n]={\overset{*}{\Lambda }}_{t}C_{N}\overset{*}{\Gamma }-\hat{L[\overset{*}{n}]}\;\Delta \psi +\left({\overset{*}{\Lambda }}_{t}C_{N}{\left({\overset{*}{\Lambda }}_{t}\right)}^{T}\right)\left[\Delta w^{\prime }+\hat{{\left(\overset{*}{r}\right)}^{\prime }}(\Delta \psi )\right],$$

(19) $$L[m]={\overset{*}{\Lambda }}_{t}C_{M}\overset{*}{K}-\hat{L[\overset{*}{m}]}\;\Delta \psi +({\overset{*}{\Lambda }}_{t}C_{M}{\left({\overset{*}{\Lambda }}_{t}\right)}^{T})\;\Delta \psi^{\prime }.$$

Here, the superscript $\overset{*}{\left(\cdot \right)}$ denotes values at previous iteration, L[·] operator denotes the linearized form of the exact equation; $L[\overset{*}{n}]$ and $L[\overset{*}{m}]$ are the converged values of linearized forces and moments obtained from the previous iteration. Δw and Δψ are the incremental displacement and rotational correction vectors, respectively. At the end of each load step, these correction vectors approach to zero and the linearized expressions $L[\overset{*}{n}]$ and $L[\overset{*}{m}]$ converge to the exact values of forces and moments given in Equations (16) and (17). The linearized counterpart of the term $\left(r^{\prime }\times n\right)$ in Equation (7) is given by,

(20) $$L[r^{\prime }\times n]=\hat{{\left(\overset{*}{r}\right)}^{\prime }}{\overset{*}{\Lambda }}_{t}C_{N}\overset{*}{\Gamma }+\left[\hat{{\left(\overset{*}{r}\right)}^{\prime }}\left({\overset{*}{\Lambda }}_{t}C_{N}{\left({\overset{*}{\Lambda }}_{t}\right)}^{T}\right)-\hat{L[\overset{*}{n}]}\right]\Delta w^{\prime }+\hat{{\left(\overset{*}{r}\right)}^{\prime }}\left[{\overset{*}{\Lambda }}_{t}C_{N}{\left({\overset{*}{\Lambda }}_{t}\right)}^{T}\hat{{\left(\overset{*}{r}\right)}^{\prime }}-\hat{L[\overset{*}{n}]}\right]\Delta \psi .$$

The details of the linearization are provided in Appendix A. The correction vectors Δw and Δψ are used to calculate the new mean line displacement vector w and the new rotation matrix Λ at the end of each Newton–Raphson iteration according to the formulas,

(21) $$w=\overset{*}{w}+\Delta w,$$

(22) $$\Lambda =\exp(\hat{\Delta \psi })\overset{*}{\Lambda },$$

where the exponentiation of the skew‐symmetric tensor $\hat{\Delta \psi }\in so(3)$ is evaluated by the Rodrigues' formula (Equation 12) to compute the rotation matrix Λ∈SO(3). This is a multiplicative and iterative approach to update the rotation matrix using the rotation matrix correction; alternatively formulations like use of total rotation vectors, (additive) incremental rotation vectors can also be found in the literature.^22^, ^30^

## 3 NUMERICAL MODEL

In this section, cell‐centered FV discretization of the computational domain and the governing balance equations is discussed. The discretization procedure is separated into two distinct parts: discretization of the solution domain and discretization of the governing equations.

### 3.1 Solution domain discretization

**FIGURE 2:**
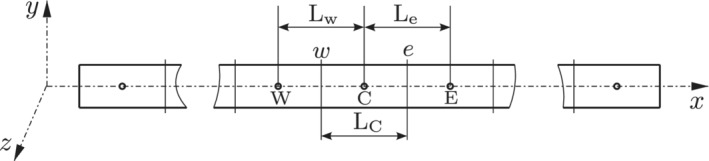
Beam body in reference configuration discretized by a finite set of 1D CVs (cells)

For the quasi‐static case, solution domain discretization implies space discretization where loads are applied gradually in pseudo‐time increments. The beam body in its reference configuration is divided into a finite number of uniform segments or control volumes (CVs) as is shown in Figure 2. A typical computational stencil (Figure 2) consists of the central CV (cell) of length $L_{C}$ with computational node C, located at the cell centroid, bounded by two internal faces w and e shared with the corresponding west and east neighboring cells, with cell centroids at W and E and lengths, $L_{w}$ and $L_{e}$ from the node C, respectively.

### 3.2 Equation discretization

**FIGURE 3:**
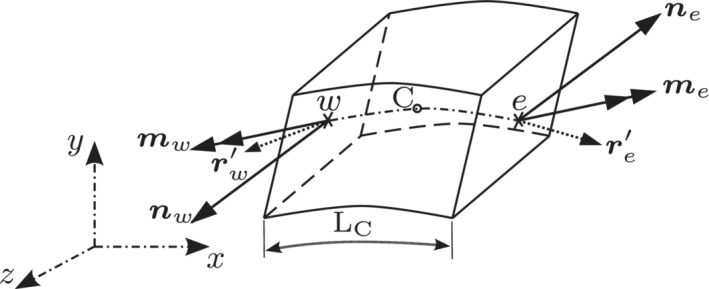
Balance of forces and moments on an isolated CV in the deformed configuration

The FV based discretization of equilibrium equations starts with a strong integral form of the governing laws, whereas, an FE approach starts with the strong differential equation, which is multiplied by an arbitrary weighting function, ϖ and integrated over material volume to obtain the equivalent weak integral form. The FV method can be retrieved from the weak form by setting ϖ=1 within the CVs and zero elsewhere.^2^ Additionally, the FV method ensures local conservation of forces, that is, forces are equal opposite across internal faces. Concretely, for an isolated CV in the deformed configuration (Figure 3), the integral form of the balance equations (Equations 8 and 9) can be discretized over a CV as following,

(23) $$\int_{L_{C}}n^{\prime }dL=n{|}_{w}^{e}=n_{e}-n_{w};\;\int_{L_{C}}f\;dL\approx f_{C}L_{C}\;\;\Rightarrow \;n_{e}-n_{w}+f_{C}L_{C}=0,$$

where $n_{e}$ and $n_{w}$ are the force values evaluated at cell faces e and w, respectively, and the subscript C represents the values at cell‐center C. The term f is assumed to have a linear variation across the CV and hence, can be approximated by the mid‐point rule.

Similarly, to discretize the moment balance equation about the cell‐center C, the first term of the moment balance equation can be exactly evaluated at cell boundaries and the integral of $\left(r^{\prime }\times n\right)$ is approximated over the CV using the trapezoidal rule and is evaluated at the faces w and e, respectively. The final discretized moment equation takes the form,

(24) $$\begin{array}{ll} & \int_{L_{C}}m^{\prime }dL=m{|}_{w}^{e}=m_{e}-m_{w};\int_{L_{C}}(r^{\prime }\times n)\;dL=\frac{1}{2}L_{C}(r_{e}^{\prime }\times n_{e})+\frac{1}{2}L_{C}(r_{w}^{\prime }\times n_{w})\\ & \;\;\Rightarrow \;m_{e}-m_{w}+\frac{1}{2}L_{C}(r_{e}^{\prime }\times n_{e})+\frac{1}{2}L_{C}(r_{w}^{\prime }\times n_{w})+t_{C}L_{C}=0. \end{array}$$

Here, t is also assumed to have a linear variation across the CV and hence is approximated by the mid‐point rule.

Unlike the traditional FE approach where the primary unknowns stored—at the nodal points—are used along with element shape functions to interpolate values to any other point, in the FV method, the unknown fields are computed at computational nodal points (cell‐centers) and interpolated, by appropriate discretization schemes, to the face centers and elsewhere. Subsequently, all the terms in the discretized equilibrium equations (Equations 23 and 24) are substituted by their linearized counterpart (Equations (18), (19), (20)).

To evaluate the internal forces and moments at cell‐faces from the linearized equations mentioned in Section 2, the mean line displacement correction vector Δw and its derivative $\Delta w^{\prime }$, and the value of cross‐section rotational correction vector Δψ and its derivative $\Delta \psi^{\prime }$ have to be approximated at the face centers in terms of the cell‐center values. All the cell‐face derivatives are approximated by the central finite difference scheme while the cell‐face values are approximated by linear interpolation. The cell‐face values on the internal faces w and e are linearly interpolated using cell‐center values as,

(25) $${\left[\left(\cdot \right)\right]}_{e}=\gamma_{e}{\left[\left(\cdot \right)\right]}_{E}+(1-\gamma_{e}){\left[\left(\cdot \right)\right]}_{C};\;{\left[\left(\cdot \right)\right]}_{w}=\gamma_{w}{\left[\left(\cdot \right)\right]}_{W}+(1-\gamma_{w}){\left[\left(\cdot \right)\right]}_{C},$$

where subscripts E and W represent values at the neighboring cell‐centers; ${\left[\left(\cdot \right)\right]}_{e}$ and ${\left[\left(\cdot \right)\right]}_{w}$ are interpolated values at internal faces e and w, respectively, and $\gamma_{e}$, $\gamma_{w}$ are the weighing factors given by,

(26) $$\gamma_{e}=\frac{1}{2}\frac{L_{C}}{L_{e}};\;\gamma_{w}=\frac{1}{2}\frac{L_{C}}{L_{w}}.$$

The notations ${\left[\left(\cdot \right)\right]}_{C}$, ${\left[\left(\cdot \right)\right]}_{W}$, and ${\left[\left(\cdot \right)\right]}_{E}$ denote the cell values at the location C and the neighboring cells W and E (Figure 2). The cell face derivatives at the internal faces w and e given by ${\left[\left(\cdot \right)\right]}_{e}^{\prime }$ and ${\left[\left(\cdot \right)\right]}_{w}^{\prime }$ are approximated using the central finite difference scheme as,

(27) $${\left[\left(\cdot \right)\right]}_{e}^{\prime }=\frac{{\left[\left(\cdot \right)\right]}_{E}-{\left[\left(\cdot \right)\right]}_{C}}{L_{e}};\;{\left[\left(\cdot \right)\right]}_{w}^{\prime }=\frac{{\left[\left(\cdot \right)\right]}_{C}-{\left[\left(\cdot \right)\right]}_{W}}{L_{w}}.$$

In the discretized equilibrium equations, all the terms are *explicitly* computed except for the primary unknowns, the correction vectors of incremental displacement and incremental rotation (Δw and Δψ), which are treated *implicitly* and are evaluated at the computational cell‐centers. As mentioned in Section 2.4, at the end of every Newton–Raphson iteration, Δw and Δψ are used to update the previously converged displacement and rotation fields (indicated by $\overset{*}{\left(\cdot \right)}$) and evaluate the deformed mean line position vector r(s) and the new rotation matrix Λ according to Equations (4), (21), and (22). The interpolated cell‐face values and their corresponding cell‐face gradients at internal faces w and e are used to compute the strain measures, Γ and K (Equations 10 and 13), which in turn are used to evaluate the spatial forces and moments at the cell‐faces (Equations 18 and 19). The employed discretization provides a nominally second order accurate approximation for displacements.

### 3.3 Initial and boundary conditions

**FIGURE 4:**
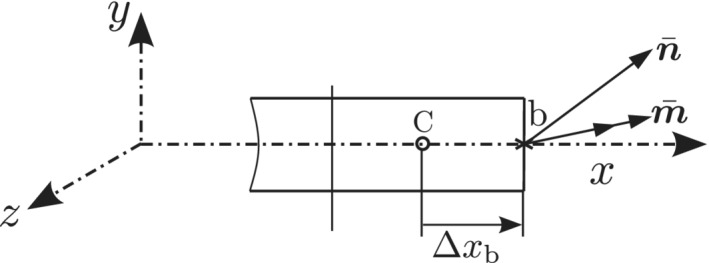
CV at the right boundary with a specified force $\bar{n}$ and moment $\bar{m}$

External forces and moments are applied in pseudo‐time increments. For the beam body, there are two boundary faces, one at the left and the other at the right of the beam. For a Dirichlet boundary condition, in the discretized governing equations (Equations 23 and 24), the displacement/rotation component at the face center of the boundary are directly replaced by the user defined value. For Neumann boundary conditions, the values of forces/moments at either boundary locations, are directly specified in Equations (23) and (24). Following solution of the linear system, the corresponding displacements and rotations at that boundary location are obtained by linear extrapolation from the interiors of the solution domain using Equations (18) and (19). For instance, Figure 4 shows the boundary face b and the neighboring cell‐center C. For a specified force $\bar{n}$ and/or moment $\bar{m}$ on the boundary, the incremental displacements/rotations can be obtained from Equations (18) and (19) as,

(28a) $$\begin{array}{ll} \bar{n} & ={\overset{*}{\Lambda }}_{t}C_{N}\overset{*}{\Gamma }+\left({\overset{*}{\Lambda }}_{t}C_{N}{\left({\overset{*}{\Lambda }}_{t}\right)}^{T}\right)\Delta w_{b}^{\prime }+\left[\left({\overset{*}{\Lambda }}_{t}C_{N}{\left({\overset{*}{\Lambda }}_{t}\right)}^{T}\right)\hat{{\left(\overset{*}{r}\right)}^{\prime }}-\hat{L[\overset{*}{n}]}\right]\Delta \psi_{b}, \end{array}$$

(28b) $$\begin{array}{ll} \bar{m} & ={\overset{*}{\Lambda }}_{t}C_{M}\overset{*}{K}-\hat{L[\overset{*}{m}]}\Delta \psi_{b}+({\overset{*}{\Lambda }}_{t}C_{M}{\left({\overset{*}{\Lambda }}_{t}\right)}^{T})\Delta \psi_{b}^{\prime }, \end{array}$$

where the cell‐face derivatives $\Delta w_{b}^{\prime }$ and $\Delta \psi_{b}^{\prime }$ are given by,

$$\begin{array}{ll} \Delta w_{b}^{\prime }=\frac{\Delta w_{b}-\Delta w_{C}}{\Delta x_{b}};\;\Delta \psi_{b}^{\prime }=\frac{\Delta \psi_{b}-\Delta \psi_{C}}{\Delta x_{b}}. & \end{array}$$

The value of $\Delta \psi_{b}^{\prime }$ is substituted into Equation (28b) and the incremental rotation vector $\Delta \psi_{b}$ is evaluated as,

$$\Delta \psi_{b}={\left(\frac{{\overset{*}{\Lambda }}_{t}C_{M}{\left({\overset{*}{\Lambda }}_{t}\right)}^{T}}{\Delta x_{b}}-\hat{L[\overset{*}{m}]}\right)}^{-1}\left[\bar{m}-{\overset{*}{\Lambda }}_{t}C_{M}\overset{*}{K}+\frac{{\overset{*}{\Lambda }}_{t}C_{M}{\left({\overset{*}{\Lambda }}_{t}\right)}^{T}}{\Delta x_{b}}\Delta \psi_{C}\right].$$

Using $\Delta \psi_{b}$ and the value of $\Delta w_{b}^{\prime }$ as shown above, the incremental displacement $\Delta w_{b}$ is then calculated as,

$$\Delta w_{b}=\Delta w_{C}+{\left(\frac{{\overset{*}{\Lambda }}_{t}C_{N}{\left({\overset{*}{\Lambda }}_{t}\right)}^{T}}{\Delta x_{b}}\right)}^{-1}\left[\bar{n}-{\overset{*}{\Lambda }}_{t}C_{N}\overset{*}{\Gamma }-[\left({\overset{*}{\Lambda }}_{t}C_{N}{\left({\overset{*}{\Lambda }}_{t}\right)}^{T}\right)\hat{{\left(\overset{*}{r}\right)}^{\prime }}-\hat{L[\overset{*}{n}]}]\Delta \psi_{b}\right].$$

### 3.4 Solution procedure

The final form of the discretized equilibrium equations, with appropriate discretization schemes described in Section 3.2, for a typical computational node C read as follows,

(29) $$A_{W}\left[\begin{array}{c} {\left(\Delta w\right)}_{W}\\ {\left(\Delta \psi \right)}_{W} \end{array}\right]+A_{C}\left[\begin{array}{c} {\left(\Delta w\right)}_{C}\\ {\left(\Delta \psi \right)}_{C} \end{array}\right]+A_{E}\left[\begin{array}{c} {\left(\Delta w\right)}_{E}\\ {\left(\Delta \psi \right)}_{E} \end{array}\right]=\left[\begin{array}{c} {\left(R^{w}\right)}_{C}\\ {\left(R^{\psi }\right)}_{C} \end{array}\right],$$

where $A_{C}$ is a coefficient matrix containing the contributions of node C while the matrices $A_{W}$ and $A_{E}$ represent the interactions of cell C with the neighboring cell centers W and E. The right‐hand side of Equation (29) is the source vector contribution. All the coefficient matrices are (6×6) dense coupled matrices with the primary unknowns being Δw and Δψ. The three components of the mean line displacement correction and cross‐section rotation vectors have to be solved in a coupled manner. The detailed structure of the diagonal and off‐diagonal coefficient matrices is provided in Appendix B.

The linearized equations (29) are assembled for all CVs forming a system of equations given by,

(30) $$\left[A\right]\left[\phi \right]=\left[R\right]$$

resulting in 6M×6M sparse matrix [A] with weak diagonal dominance, where M is the total number of CVs. The coefficients $A_{C}$ constitute the diagonal of [A] whereas matrices $A_{W}$ and $A_{E}$ contribute to its off‐diagonal terms. The solution vector $\left[\phi \right]$ contains the primary unknowns Δw and Δψ, and $\left[R\right]$ is the source vector containing the explicit discretized terms and boundary condition contributions. The final system of linearized algebraic equations, obtained by assembling Equation (29) for all control volumes in the mesh, is successfully solved using the block variant of the Thomas algorithm.

Algorithm 1 **Solution procedure; ${\left(\cdot \right)}_{f}$: fields at the face centers**

1

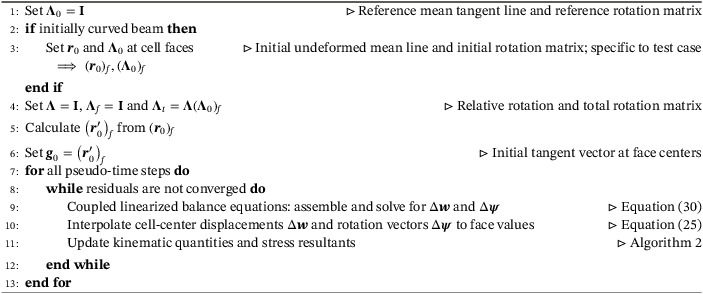

For every pseudo‐time increment, the coupled equations are iteratively solved by Newton–Raphson procedure, until a user‐defined convergence tolerance is achieved with the maximum number of allowable iterations set to 30. For convergence, both the Euclidean norms of the solution increment vectors (‖ϕ‖) and residuals from the linear system of equations (‖R‖) are checked. Since the displacements are additively updated and the rotations in a multiplicative manner, the norms of displacement correction vector (‖Δw‖) and the rotation correction vector ‖Δψ‖ are separately calculated and the solution increment residual is set as ‖ϕ‖=max(‖Δw‖,‖Δψ‖). For convergence of the solver, after each iteration, either of the two norms have to fall below a prescribed tolerance, that is, $\left\|R\|<(\delta_{R}=10^{-6}\right)$ and $\left\|\phi \|<(\delta_{\phi }=10^{-10}\right)$. The current method has been implemented in open‐source software OpenFOAM^42^ (version foam‐extend‐4.1), exploiting the developed object oriented FV procedures. The overall solution procedure is summarized in Algorithm 1 and the procedure to update the kinematic quantities and stress resultants is given in Algorithm 2.

Algorithm 2 **Update of the kinematic quantities and stress resultants; ${\left(\cdot \right)}_{f}$: fields at the face centers, $\overset{*}{\left(\cdot \right)}$: fields calculated in the previous Newton–Raphson iteration**

1

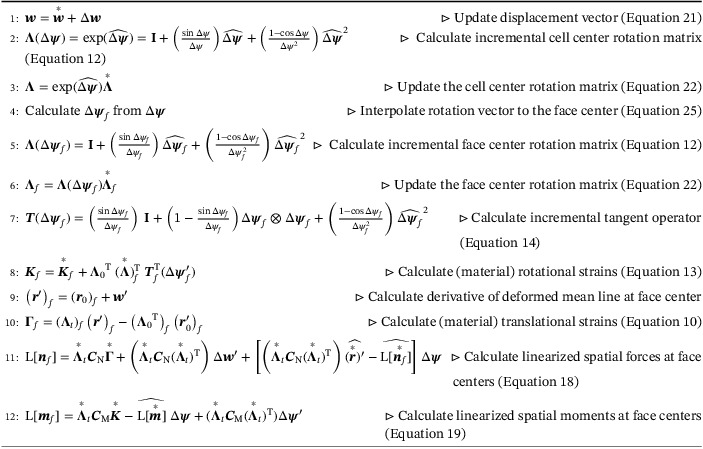

## 4 VERIFICATION TEST CASES

In this section, the capabilities of the developed FV methodology are investigated in the five complementary benchmark cases:

1. Rigid rotation of an initially curved beam: this case verifies the objectivity of the adopted strains and rotation interpolation measures.
2. In‐plane bending of a cantilever beam subjected to a concentrated moment at one end: this case provides an analytical solution to check the accuracy and order of accuracy of the proposed methodology. A subsection of this test case is also used to demonstrate the ability of the FV methodology to avoid shear locking effects.
3. Out‐of‐plane bending of the cantilever to form a helix due to a concentrated moment and an out‐of‐plane force: this case with complex loading conditions, tests the ability of the numerical solver to cater for large rotations and 3D deformation.
4. A cantilever, initially bent into a $45^{\circ }$ arc in xy‐plane, subjected to a force along the z‐direction: this is another benchmark curved beam case used by many authors to establish the accuracy and order of accuracy of the numerical model.
5. Deep‐circular arch with a concentrated in‐plane force at the crown location: this unsymmetrical circular arch case has exact solutions available and is used to assess the ability of the solver to obtain the critical buckling load value.

All the test cases have been executed using a quad‐core CPU with hyper‐threading (Intel Core(TM) i7‐8565U CPU with base frequency 1.80 GHz and maximum turbo frequency 4.6 GHz).

### 4.1 Rigid rotation of an initially curved beam

**FIGURE 5:**
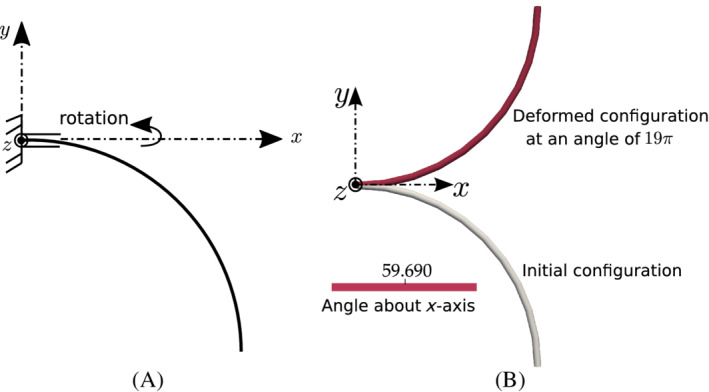
Rigid rotation of curved cantilever: (A) Test case setup and (B) deformed configuration of the beam for a rotation angle, $\psi_{x}=19\pi$

For the FV formulation presented in this article and the type of the rotation interpolation adopted, the objectivity of the conjugate strain measures is verified using the numerical test case first presented in Meier et al.^1^, ^40^ as shown in Figure 5A. For an initially curved beam with a centerline configuration of a quarter circle with radius R=100 m, initial length, L=πR/2=157.079 m, discretized into 10 CVs, a rotation of 20π is gradually applied about the global x‐axis in 100 load increments. The mechanical properties for this test case are, E=1 GPa and G=0.5 GPa. The nature of the boundary condition at the clamped left end ($w_{x}=w_{y}=w_{z}=0$ and $\psi_{y}=\psi_{z}=0$) of the beam is such that, this prescribed rotation should only cause rigid body rotation in the beam about the x‐axis without any deformation and no accumulation of internal strain energy should be observed.

Figure 5B shows the deformed beam configuration for an angle $\psi_{x}=19\pi$ about the x‐axis and it is evident that the deformed beam is rigidly rotated without any unphysical deformation. For the test case, execution time is approximately 1.5 s and requires 3 outer iterations on average to converge per pseudo‐time increment. For each CV, the strains Γ and K are used to calculate the internal energy as per Equation (15) and summed over all the CVs to obtain the total accumulated strain energy of the beam due to the applied rotation. The total energy is found to be zero to machine precision (in the order of $10^{-31}$) and hence, the objectivity of the adopted strain measures is confirmed.

### 4.2 In‐plane bending of a cantilever beam subjected to a concentrated moment at one end

This in‐plane pure flexural bending of a cantilever case has been investigated by a number of authors.^18^, ^22^ An initially straight cantilever of length L=10 m is bent into a circle by applying a concentrated moment at one end. The mechanical properties available in the literature for this test case are $EA=10^{4}$ N , $GA_{2}=GA_{3}=5000$ N, $EI_{2}=EI_{3}=100$ ${\text{N m}}^{2}$, GJ=100 ${\text{N m}}^{2}$, respectively. Hence, the cross‐section radius of the beam, r=0.2 m and the Young's modulus $E=7.95\times 10^{4}$ Pa are assumed in a way to achieve these desired numerical values. The Poisson's ratio ν is taken as zero. According to the classic Euler formula, the analytical solution for pure beam‐bending is given by,

(31) $$\psi_{z}=\frac{M_{z}L}{EI};\;w_{x}=L-\frac{L}{\psi_{z}/2}\sin\frac{\psi_{z}}{2}\cos\frac{\psi_{z}}{2};\;w_{y}=\frac{L}{\psi_{z}/2}{\left(\sin\frac{\psi_{z}}{2}\right)}^{2},$$

where $\psi_{z}$, $w_{x}$, and $w_{y}$ are the rotation and in‐plane displacements of the beam tip.

**FIGURE 6:**
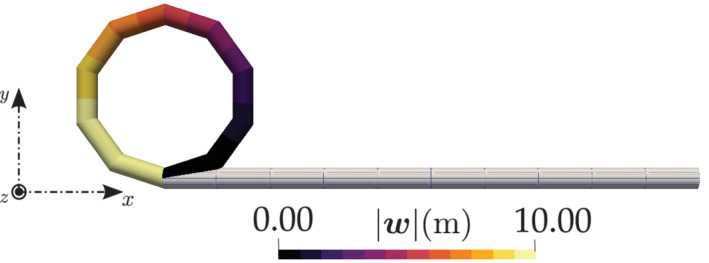
In‐plane bending of a cantilever beam subjected to concentrated moment at one end: Deformed configuration for $\psi_{z}=2\pi$

**FIGURE 7:**
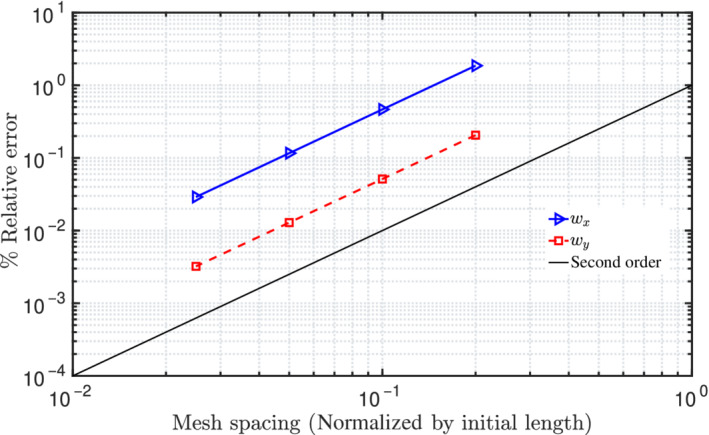
In‐plane bending of a cantilever beam subjected to concentrated moment at one end: Mesh convergence plot of in‐plane displacements for an applied moment M=2.5π N m

To compare the results of this test case with the ones available in literature,^18^ the computational domain is discretized into 5 CVs and a moment of $M_{z}=20\pi$ N m $\left(\psi_{z}=2\pi \right)$ is applied at the right end in one load step and the Newton–Raphson solution procedure converges in 2 outer iterations. The equation and solution increment residuals per iteration are shown in Table 1. Figure 6 shows the deformed configuration of the cantilever for 10 CVs and a moment value of 20π N m. For an applied moment, $M_{z}=2.5\pi$ N m and a mesh of 5 CVs, the tip in‐plane displacements are found to be $w_{x}=-1.00146$ m and $w_{y}=3.72731$ m, respectively, which vary from the analytical solution (Equation 31) by only 0.4% and 0.05%, respectively. For comparing the numerically obtained results with the reference, a percentage relative error is calculated as,

(32) $$\text{\% Relative error}=\left|\frac{\xi^{\text{num}}-\xi^{\text{ref}}}{\xi^{\text{ref}}}\right|\times 100\%,$$

where $\xi^{\text{num}}$ denotes the numerical value and $\xi^{\text{ref}}$ is the reference/analytical result. Figure 7 shows the percentage mesh error convergence of the in‐plane displacements for successive reduction of mesh sizes, that is, 5, 10, 20, and 40 CVs; a quadratic order of error convergence is observed.

**TABLE 1:** In‐plane bending of cantilever: Residual values for the single load step

Iter. #	‖ϕ‖	‖R‖
0	$2.51\times 10^{1}$	$6.28\times 10^{2}$
1	$2.71\times 10^{1}$	$1.38\times 10^{5}$
2	$5.79\times 10^{-11}$	$2.02\times 10^{-7}$

**FIGURE 8:**
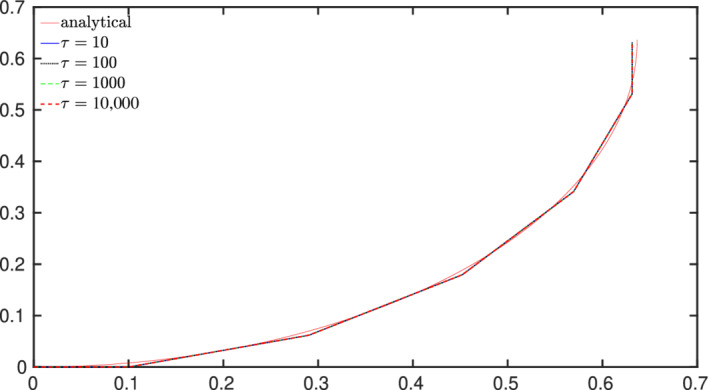
Pure bending of cantilever beam: Deformed shape of beam centerline for different τ values

*Shear locking study*. The same test case of in‐plane bending of a cantilever is used with mechanical properties adopted from Meier et al.^1^ to study shear locking phenomenon. This representative test case suffers from shear locking if a fully integrated FE formulation is used. A beam of square cross‐section (side‐length a), length L=1 m, divided into 5 CVs, is fixed at the left end and a discrete moment, $M_{z}=0.5\pi EI_{zz}/L$ is applied at the right end in one load step. The analytical solution for this test case is the beam centerline deformed into a quarter circle. The mechanical properties are set as, Young's modulus, E=1 Pa and Poisson's ratio ν=0 and the test case is simulated for different slenderness ratios $\left(\tau =\frac{L}{a}\right)$, that is, τ=10, τ=100, τ=1000, and τ=10,000. The external moment is adapted for each value of τ such the deformed line represents the same quarter circle. The deformed centerline of the beam for all τ values is presented in Figure 8. The solver converges in three outer iterations for all τ values. It is evident from the figure that shear‐locking is not observed for increasing τ values.

The shear locking phenomenon mechanically leads to accumulation of parasitic stress and results in an overly stiff system. Meier et al.^1^ adopted a technique of calculating the constraint ratio (r) of a problem that provides a heuristic evaluation of the locking phenomenon. The constraint ratio is defined as the ratio of the total number of equilibrium equations $n_{eq}$ to the total number of constraint equations $n_{eq,c}$. If the constraint ratio of the discretized problem ($r_{h}$) is greater than the constraint ratio (r) of the space‐continuous problem, the system usually tends to lock. The Simo–Reissner based beam formulation requires 6 equilibrium equations ($n_{eq}=6$) to describe the problem. For the pure bending case, the vanishing shear and axial strains (Γ=0) lead to $n_{eq,c}=3$ constraint equations and hence, $r=\frac{n_{eq}}{n_{eq,c}}=2$. For the discretized problem with p CVs, total equilibrium conditions are $n_{eq}=6p$ and total constraint equations are $n_{eq,c}=3p$, which yields $r_{h}=2$. Since, $r_{h}=r$ (optimal constraint ratio), no shear‐locking phenomenon should be observed and that is precisely the observation from the simulated test case (Figure 8). For the FE formulation of Simo–Reissner beams, techniques like reduced integration have to be adopted to ensure optimal constraint ratio and avoid locking effects.^1^ Since the FV formulation is analogous to the reduced integration FE approach, it intrinsically avoids the shear‐locking effects without compromising the (second‐order) accuracy in predicting displacements and forces.

### 4.3 Out‐of‐plane bending of the cantilever to form a helix due to a concentrated moment and an out‐of‐plane force

For this case, the previous problem is extended by applying a concentrated out‐of‐plane force at the free end of the beam, n=[0,0,50] N along with a rotation $\psi_{z}=20\pi$ (m=[0,0,200π] N m) about the z‐axis. Thus, an initially straight cantilever beam is bent to a circular helix shape. The constitutive matrices are taken similar to the previous case, that is, $C_{N}=\text{diag}[10^{6},5\times 10^{5},5\times 10^{5}]$ N and $C_{M}=\text{diag}[10^{2},10^{2},10^{2}]$ N m${}^{2}$, diag[] denotes a diagonal matrix. The cross‐section radius is reduced to r=0.02 m and the Young's modulus is taken as, $E=7.95\times 10^{8}$ Pa, so that the beam does not overlap into its own body while forming the helix. For comparing the achieved numerical results with those published by Ibrahimbegovic,^20^ the beam of initial length L=10 m is discretized into 100 uniform CVs.

**FIGURE 9:**
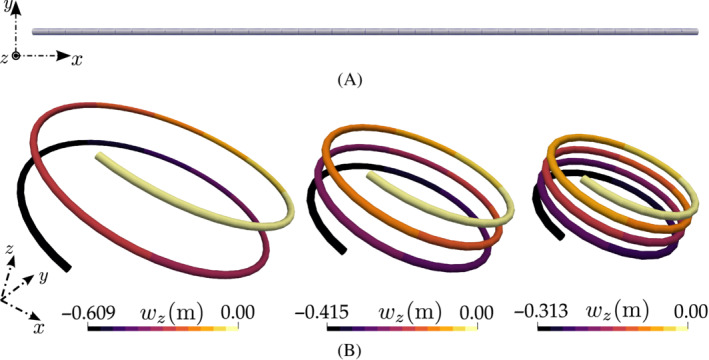
Out‐of‐plane bending of the cantilever to form a helix due to a concentrated moment and an out‐of‐plane force: (A) Initial beam configuration and (B) deformed beam for 20% (left), 30% (middle), and 40% (right) of the applied load

**FIGURE 10:**
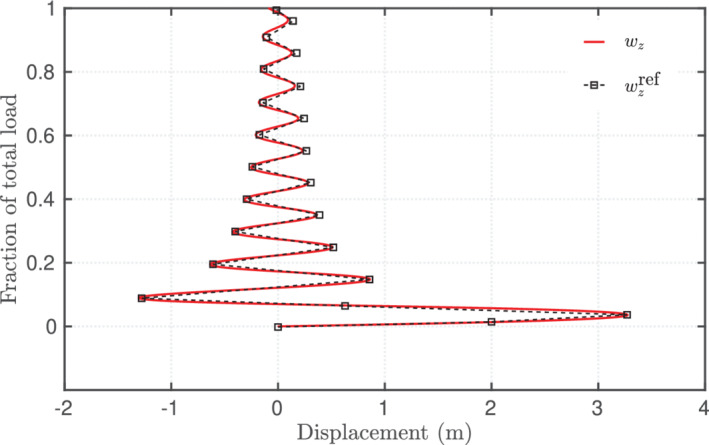
Out‐of‐plane bending of the cantilever to form a helix due to a concentrated moment and an out‐of‐plane force: Free‐end displacement component in the direction of applied load ($w_{z}^{\text{ref}}$ adapted from Reference 20)

The external loads to the beam are applied in 1000 pseudo‐time increments and the final load is reached at a pseudo time‐value of 10 s. The Newton–Raphson solution procedure required 4 outer iterations on average to converge per pseudo time increment and 12 s of total execution time. The equation and solution increment residuals for all the iterations in the last load step are presented in Table 2. Figure 9A shows the initial beam and Figure 9B shows the deformed shape of the beam for 20%, 30%, and 40% of the total loading, respectively. Figure 10 presents the free‐end displacement component $w_{z}$ as a function of the applied load. From Figure 10, it is evident that with increasing rotations, the out‐of‐plane displacement oscillates about the z‐axis crossing the zero value. This ability of the solver to capture the complex deformation and oscillatory motion of the beam is possible because of the incremental rotation vector‐based parameterization and the results (Figure 10) are in good agreement with those published by Ibrahimbegovic;^20^ note the same test case failed to converge when a non‐incremental formulation was initially trialed.

**TABLE 2:** Out‐of‐plane bending of cantilever into helix: Residuals for the last load step (pseudo time increment, Δt=0.01 s)

Iter. #	‖ϕ‖	‖R‖
0	$6.29\times 10^{-2}$	$6.30\times 10^{1}$
1	$3.33\times 10^{-4}$	$5.73\times 10^{3}$
2	$5.72\times 10^{-5}$	$3.72\times 10^{-2}$
3	$7.19\times 10^{-10}$	$3.68\times 10^{-4}$
4	$2.59\times 10^{-12}$	$5.38\times 10^{-8}$

### 4.4 A cantilever, initially bent into a $45^{\circ }$ arc in xy‐plane, subjected to a force along the z‐direction

This case consists of an initially curved cantilever beam with an applied vertical concentrated force along z‐axis. Several authors have reproduced this benchmark case.^18^, ^22^, ^29^, ^30^, ^43^ The cantilever, which has a unit square cross‐section, is initially bent into a $45^{\circ }$ arc of radius 100 m, length L=πR/4=78.54 m and then a vertical force, $n_{z}=600$ N is applied. The mechanical properties adopted are $E=1\times 10^{7}$ Pa and ν=0. For a spatial discretization of 8 CVs, the tip end displacements values are $w_{x}=-23.5281$ m, $w_{y}=-13.5415$ m, and $w_{z}=53.082$ m, respectively. Simo and Vu‐Quoc^18^ apply a load in three steps (300, 150, and 150 N) and take 27 cumulative iterations to attain convergence; the current model takes 19 cumulative iterations for the same loading increment. The comparison of iterations per load step for the current model and the position of the tip geometry are compared with those reported by Simo and are presented in Table 3. On the other hand, for a systematic loading in six equal load steps, it takes an average of 5 outer iterations to converge per pseudo time increment.

**TABLE 3:** Comparison of the tip geometry and the iterations per load value of the initially curved cantilever test case with literature

	Simo and Vu‐Quoc^18^				Current FV model			
Load (N)	Iter #	x	y	z	Iter #	x	y	z
300	13	58.84	22.33	40.08	7	58.832	22.305	39.879
450	8	52.32	18.62	48.39	6	52.284	18.576	48.146
600	6	47.23	15.79	53.37	6	47.179	15.754	53.217

**FIGURE 11:**
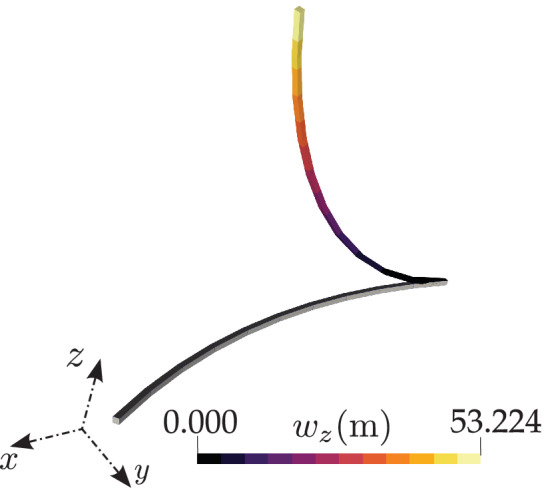
A cantilever, initially bent into a $45^{\circ }$ arc in xy‐plane, subjected to a force along the z‐direction: Initial and deformed configuration for an applied load of 600 N

For this test case, the reported displacements in the literature are for a discretization of 8 linear FE elements and the current numerical results (for 10 CVs) are found to be comparable with the ones presented in the literature (Table 4). Figure 11 shows the initial and the deformed configuration of the beam and the displacement component $w_{z}$ for 10 CVs. This test case provides a true 3D setting to test the convergence of the Newton–Rapshon solution procedure. To that end, when the total load of 600 N is applied in six equal load steps, the convergence rate of the Newton–Raphson solver for the second and final load step is shown in Table 5. The results reported by Ibrahimbegović^22^ state that their model takes about nine iterations in for the second load step to achieve a residual convergence (‖R‖) tolerance below $10^{-8}$, whereas the current model takes six iterations to converge. Furthermore, if the total load of 600 N is applied in a single load step, it is observed that the quadratic convergence of the Newton–Raphson solver is lost and the solver converges in nine iterations.

**TABLE 4:** Displacement values observed for a curved cantilever in xy‐plane subjected to a force along z‐axis

Numerical results	$\left|w_{x}\right|$	$\left|w_{y}\right|$	$\left|w_{z}\right|$
Present	23.540	13.564	53.310
Bathe and Bolourchi^43^	23.5	13.4	53.4
Simo and Vu‐Quoc^18^	23.48	13.50	53.37
Cardona and Geradin^30^	23.67	13.73	53.50
Crisfield^29^	23.87	13.63	53.71
Ibrahimbegović^22^	23.697	13.668	53.498

**TABLE 5:** A cantilever, initially bent into a $45^{\circ }$ arc in xy‐plane: Residuals for the second and final load step

Second load step ($n_{z}=200$ N)		Final load step	
Iter #	Residuals	Iter #	Residuals
0	15.2704	0	5.028
1	2.3932	1	$5.418\times 10^{-1}$
2	$4.967\times 10^{-2}$	2	$6.085\times 10^{-3}$
3	$2.572\times 10^{-3}$	3	$1.812\times 10^{-4}$
4	$5.229\times 10^{-7}$	4	$4.793\times 10^{-8}$
5	$3.988\times 10^{-9}$	5	$2.225\times 10^{-11}$
6	$2.895\times 10^{-15}$		

**FIGURE 12:**
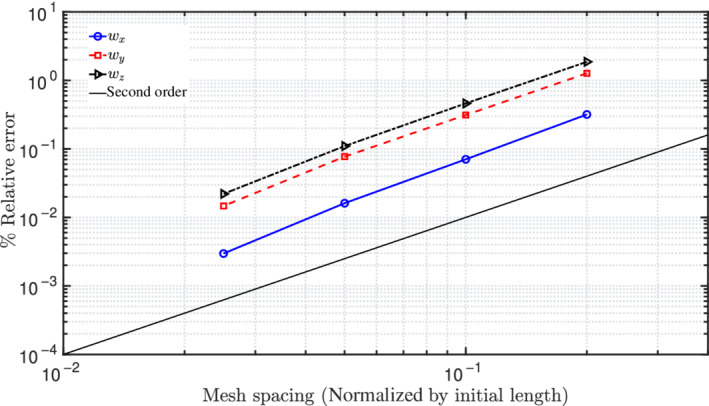
A cantilever, initially bent into a $45^{\circ }$ arc in xy‐plane, subjected to a force along the z‐direction: mesh convergence of the displacement components for the applied load, $n_{z}=600$ N

**FIGURE 13:**
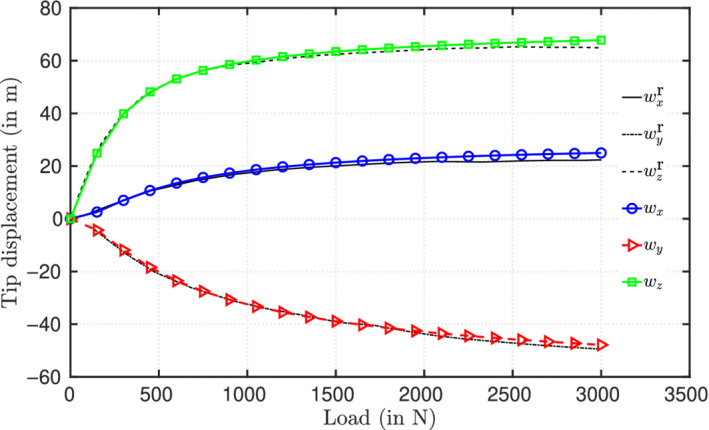
A cantilever, initially bent into a $45^{\circ }$ arc in xy‐plane, subjected to a force of 3000 N along the z‐direction: The end‐tip displacements for increasing load values

Figure 12 presents the % mesh error convergence of the displacement components for successive mesh size reductions, namely, 5,10,20, and 40 CVs; a quadratic order of error convergence is observed. The analytical solutions for this test case are not available and the numerical results reported in literature are similar. To calculate the % mesh discretization error (Equation 32), the displacement values corresponding to a finer mesh of 80 CVs are adopted as reference results here. For comparing the displacement versus load curves presented in Simo et al.,^18^ the beam is discretized into 8 CVs and an end force of 3000 N is applied to its free end at 30 N load increments. Figure 13 presents the displacement components of the free end of the beam and the results are found to be in close agreement with those reported by Simo et al.^18^

**FIGURE 14:**
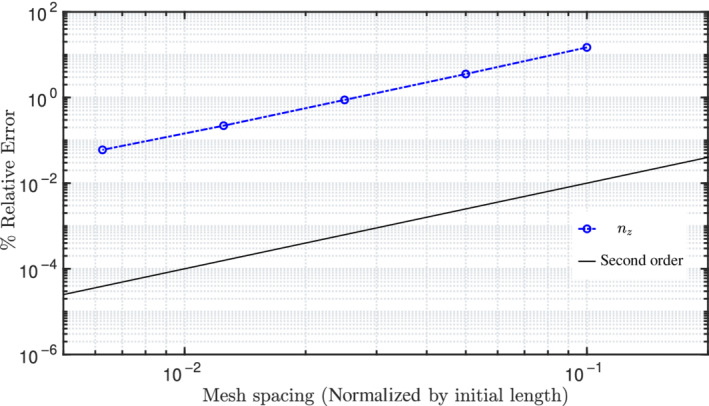
A cantilever, initially bent into a $45^{\circ }$ arc in xy‐plane: Mesh convergence of the force $n_{z}$ for an applied displacement, $w_{x}=-23.5607$ m, $w_{y}=-13.6048$ m, $w_{z}=53.4756$ m

To investigate the order of accuracy of the solver in calculating forces, the present test case is reproduced by applying displacements at the right end instead of forces. To obtain mesh independent displacements, a fine mesh of 1280 CVs is selected and for an applied force of 600 N along the z‐axis, the values observed are $w_{x}=-23.5607$ m, $w_{y}=-13.6048$ m, $w_{z}=53.4756$ m. On applying these displacements to the same test case, the exact force vector (00600) N should be retrieved. Consequently, the test case is run by applying the displacements (−23.5607,−13.6048,53.4756) m for different mesh sizes, namely, 10, 20, 40, 80, and 160 CVs. Figure 14 shows the % mesh error convergence of the vertical force ($n_{z}$) for successive mesh size reductions; a quadratic order of error convergence is observed. Since the true value of forces $n_{x}$ and $n_{y}$ expected for the given displacements is zero, relative error is not defined; however, the force components obtained for successive mesh reductions are seen to go to zero (Table 6).

**TABLE 6:** Curved cantilever in xy‐plane subjected to a force along z‐axis: Force values observed for different mesh sizes

CVs	$n_{x}$ (N)	$n_{y}$ (N)
10	53.98	24.76
20	12.81	5.91
40	3.17	1.47
80	0.80	0.37
160	0.21	0.09

### 4.5 Deep‐circular arch with a concentrated in‐plane force at the crown location

The instabilities of asymmetric clamped‐hinged arches were first investigated by DaDeppo and Schmidt^44^ and then by many other authors.^18^, ^40^, ^45^ In the current work, a $215^{\circ }$ deep circular arch with a unit circular cross‐section, hinged at the left end ($w_{x}=w_{y}=w_{z}=0$) and clamped at the right ($w_{x}=w_{y}=w_{z}=0$ and $\psi_{x}=\psi_{y}=\psi_{z}=0$), having a radius R=100 m and length 375.245 m is considered and a force‐displacement behavior of the arch is studied until buckling. A concentrated point load $P\equiv (0\;n_{y}\;0)$ acts at the crown location of the arch. The adopted mechanical properties are: $EI_{2}=EI_{3}=GJ=1\times 10^{4}$ N m${}^{2}$.

**FIGURE 15:**
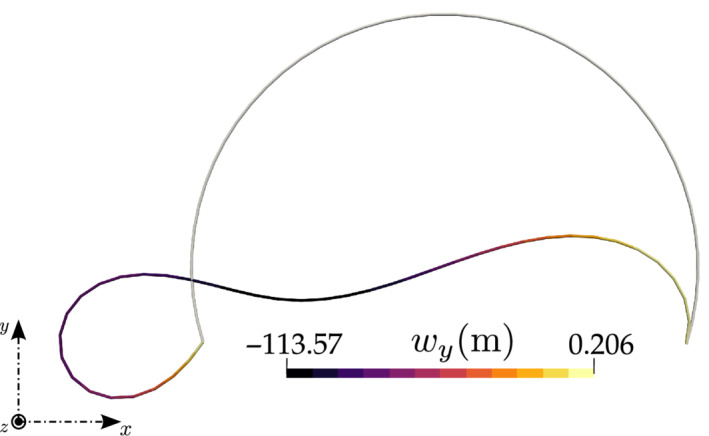
Deep‐circular arch with a concentrated in‐plane force at the crown location: Initial and deformed configuration

**FIGURE 16:**
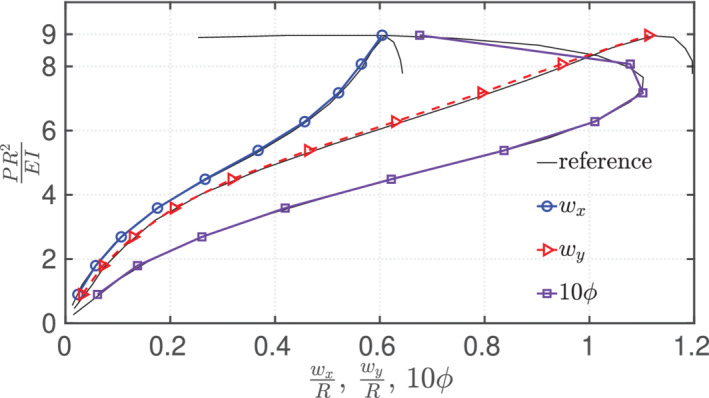
Deep‐circular arch with a concentrated in‐plane force at the crown location: The normalized horizontal and vertical displacements of the crown point ($w_{x}/R,w_{y}/R$) and the rotation angle (10ϕ) of the crown point for different loading values of the force

In accordance with the literature,^18^ the entire spatial domain is discretized into 40 CVs and Figure 15 shows the initial and the deformed configuration of the arch. For an increasing load at 1 N increments up to 8 N and 0.005 N increments beyond 8 N , the predictive critical buckling load is found to be 9.065 N; the exact value reported by DaDeppo and Schmidt^44^ is 8.97 N. The solver, being quasi‐static, does not converge for the unstable post‐buckling analysis of the circular arch; beyond the critical buckling load, dynamic analysis or special procedures would be required. For the load steps with force increments of 1 N until 8 N, the residuals converge at an average of 4 iterations per pseudo time increment; for loads higher than 8 N, per 0.005 N force increment, an average of 3 iterations are required to converge until the critical buckling load of 9.065 N is achieved. The total time of computation is less than 2 s. Figure 16 shows the variation of the dimensionless quantity $\frac{PR^{2}}{EI}$ versus the normalized displacements ($w_{x}$ and $w_{y}$ with respect to R) and rotation angle ϕ of the tangent vector at the crown location. The results are found to be in good agreement with the exact solutions presented by DaDeppo and Schmidt.^44^

## 5 CONCLUDING REMARKS

This article is the first to develop and verify a total Lagrangian cell‐centered finite volume methodology for geometrically exact beams with arbitrary initial curvatures subjected to finite displacements and rotations. The mathematical formulation and the corresponding FV‐based spatial and equation discretization are described in detail. The potential of the developed methodology has been tested using five complementary benchmark test cases, where the spatial discretization has been shown to be second‐order accurate for displacements and forces. For the cases examined, the numerical results obtained using the FV technique have been found to be in good agreement with the analytical results and FE based numerical results reported in the literature. In the $45^{\circ }$ curved cantilever test case (Section 4.4), for unequal load increment values ($n_{z}=300$, 450, and 600 N), the current FV formulation requires 19 cumulative Newton–Raphson iterations to converge in comparison to the 27 total iterations as reported by Simo et al.^18^ Moreover, the nature of the FV formulation intrinsically avoids the locking effects observed in general fully integrated FE based formulations and also ensures exact conservation of moments and forces at a local as well as global level. Extension of the quasi‐static FV formulation to dynamic analysis will be considered in future work.

## CONFLICT OF INTEREST

The authors declare that they have no conflict of interest.

## APPENDIX A LINEARIZATION OF STRESS‐RESULTANTS

The linearization of the stress‐resultants involves a systematic use of the directional derivatives of the kinematic quantities and the strain measures. For a given beam configuration, the linearization of perturbed kinematic quantities $\left(r_{\epsilon }=r_{0}+w+\epsilon \Delta w,{\left(\Lambda_{t}\right)}_{\epsilon }=\exp(\epsilon \hat{\Delta \psi })\Lambda_{t}\right)$ yields,

(A1)
$$\frac{d}{d\epsilon }\left(r_{\epsilon }\right){|}_{\epsilon =0}=\Delta w,\;\frac{d}{d\epsilon }\left({\left(\Lambda_{t}\right)}_{\epsilon }\right){|}_{\epsilon =0}=\hat{\Delta \psi }\;\Lambda_{t},$$

where ϵ is a small scalar perturbation. The linearized forms for the perturbed (material) strain measures $\Gamma_{\epsilon }={\left(\Lambda_{t}^{T}\right)}_{\epsilon }r_{\epsilon }^{\prime }-e_{1}$ and $K_{\epsilon }={\left(\Lambda_{t}^{T}\right)}_{\epsilon }{\left(\Lambda_{t}^{\prime }\right)}_{\epsilon }$ ^18^ are,

(A2)
$$\frac{d}{d\epsilon }\left(\Gamma_{\epsilon }\right){|}_{\epsilon =0}={\left(\Lambda_{t}\right)}^{T}\hat{{\left(r\right)}^{\prime }}(\Delta \psi )+{\left(\Lambda_{t}\right)}^{T}\Delta w^{\prime },$$

(A3)
$$\frac{d}{d\epsilon }\left(K_{\epsilon }\right){|}_{\epsilon =0}={\left(\Lambda_{t}\right)}^{T}\Delta \psi^{\prime }.$$

Using these linearized strain measures, the spatial force, $n=\Lambda_{t}C_{N}\Gamma$, upon linearization yields,

(A4a)
$$L[n]={\overset{*}{\Lambda }}_{t}C_{N}\overset{*}{\Gamma }+\frac{d}{d\epsilon }\left({\left(\Lambda_{t}\right)}_{\epsilon }C_{N}\Gamma_{\epsilon }\right){|}_{\epsilon =0},$$

where $\overset{*}{\left(\cdot \right)}$ denotes the *known* fields from the previously converged Newton–Raphson iteration. The first term in the RHS of the above equation is the explicit (*unbalanced*) force evaluated using the previous converged values of kinematic quantities and strains; the second part of the equation is the directional derivative of force, which on further expansion leads to,

(A4b)
$$\begin{array}{ll} \;\Rightarrow \;L[n] & ={\overset{*}{\Lambda }}_{t}C_{N}\overset{*}{\Gamma }+\frac{d}{d\epsilon }{\left(\Lambda_{t}\right)}_{\epsilon }{|}_{\epsilon =0}C_{N}\Gamma +(\Lambda_{t})C_{N}\frac{d}{d\epsilon }(\Gamma_{\epsilon }){|}_{\epsilon =0}, \end{array}$$

(A4c)
$$\begin{array}{ll} & ={\overset{*}{\Lambda }}_{t}C_{N}\overset{*}{\Gamma }+\hat{\Delta \psi }\;\left(\Lambda_{t}C_{N}\Gamma \right)+\left(\Lambda_{t}C_{N}{\left(\Lambda_{t}\right)}^{T}\right)\left[\Delta w^{\prime }+\hat{{\left(r\right)}^{\prime }}\;(\Delta \psi )\right]. \end{array}$$

In Equation (A4c), the coefficient attached to $\hat{\Delta \psi }$ in the second term is also “force,” which is substituted by the “linearized force” calculated from the previous iteration and, for all the other fields appearing in the above equation, known values from previous iteration are used. Hence, the final form of the linearized spatial force is given as,

(A4d)
$$\;\Rightarrow \;L[n]={\overset{*}{\Lambda }}_{t}C_{N}\overset{*}{\Gamma }-\hat{L[\overset{*}{n}]}\;\Delta \psi +\left({\overset{*}{\Lambda }}_{t}C_{N}{\left({\overset{*}{\Lambda }}_{t}\right)}^{T}\right)\left[\Delta w^{\prime }+\hat{{\left(\overset{*}{r}\right)}^{\prime }}\;(\Delta \psi )\right].$$

Similarly, the spatial moment, $m=\Lambda_{t}C_{M}K$ can be linearized as,

(A5)
$$\begin{array}{ll} L[m] & ={\overset{*}{\Lambda }}_{t}C_{M}\overset{*}{K}+\frac{d}{d\epsilon }\left({\left(\Lambda_{t}\right)}_{\epsilon }C_{M}K_{\epsilon }\right){|}_{\epsilon =0}\\ & ={\overset{*}{\Lambda }}_{t}C_{M}\overset{*}{K}+\frac{d}{d\epsilon }{\left(\Lambda_{t}\right)}_{\epsilon }{|}_{\epsilon =0}C_{M}K+\Lambda_{t}C_{M}\frac{d}{d\epsilon }(K_{\epsilon }){|}_{\epsilon =0}\\ \;\Rightarrow \;L[m] & ={\overset{*}{\Lambda }}_{t}C_{M}\overset{*}{K}-\hat{L[\overset{*}{m}]}\;\Delta \psi +({\overset{*}{\Lambda }}_{t}C_{M}{\left({\overset{*}{\Lambda }}_{t}\right)}^{T})\;\Delta \psi^{\prime }. \end{array}$$

The linearized counterpart of the term $\left(r^{\prime }\times n\right)$ in Equation (7) is given by,

(A6)
$$\begin{array}{ll} L[r^{\prime }\times n] & ={\left(\overset{*}{r}\right)}^{\prime }\times ({\overset{*}{\Lambda }}_{t})C_{N}\overset{*}{\Gamma }+\frac{d}{d\epsilon }\left(r_{\epsilon }^{\prime }\times {\left(\Lambda_{t}\right)}_{\epsilon }C_{N}\Gamma_{\epsilon }\right){|}_{\epsilon =0}\\ & ={\left(\overset{*}{r}\right)}^{\prime }\times ({\overset{*}{\Lambda }}_{t})C_{N}\overset{*}{\Gamma }+\frac{d}{d\epsilon }(r_{\epsilon }^{\prime }){|}_{\epsilon =0}\times (\Lambda_{t}C_{N}\Gamma )\\ & \;+{\left(r\right)}^{\prime }\times \frac{d}{d\epsilon }\left({\left(\Lambda_{t}\right)}_{\epsilon }\right){|}_{\epsilon =0}C_{N}\Gamma +{\left(r\right)}^{\prime }\times \Lambda_{t}C_{N}\frac{d}{d\epsilon }\left(\Gamma_{\epsilon }\right){|}_{\epsilon =0}\\ & ={\left(\overset{*}{r}\right)}^{\prime }\times ({\overset{*}{\Lambda }}_{t})C_{N}\overset{*}{\Gamma }+\Delta w^{\prime }\times L[\overset{*}{n}]+{\left(\overset{*}{r}\right)}^{\prime }\times \left(\hat{\Delta \psi }\right)\;L[\overset{*}{n}]\\ & \;+{\left(\overset{*}{r}\right)}^{\prime }\times {\overset{*}{\Lambda }}_{t}C_{N}\left[{\left({\overset{*}{\Lambda }}_{t}\right)}^{T}\hat{{\left(\overset{*}{r}\right)}^{\prime }}\;(\Delta \psi )+{\left({\overset{*}{\Lambda }}_{t}\right)}^{T}\Delta w^{\prime }\right]\\ \;\Rightarrow \;L[r^{\prime }\times n] & =\hat{{\left(\overset{*}{r}\right)}^{\prime }}\;({\overset{*}{\Lambda }}_{t}C_{N}\overset{*}{\Gamma })+[\hat{{\left(\overset{*}{r}\right)}^{\prime }}\left({\overset{*}{\Lambda }}_{t}C_{N}{\left({\overset{*}{\Lambda }}_{t}\right)}^{T}\right)-\hat{L[\overset{*}{n}]}]\Delta w^{\prime }\\ & \;+\hat{{\left(\overset{*}{r}\right)}^{\prime }}[{\overset{*}{\Lambda }}_{t}C_{N}{\left({\overset{*}{\Lambda }}_{t}\right)}^{T}\hat{{\left(\overset{*}{r}\right)}^{\prime }}-\hat{L[\overset{*}{n}]}]\Delta \psi . \end{array}$$

The manipulation of skew‐symmetric tensors and the corresponding axial vector relation, $\theta \times h=\hat{\theta }h=-(h\times \theta )=-\hat{h}\theta$ is used in all the above expressions.

## APPENDIX B COEFFICIENTS OF THE BLOCK LINEAR SYSTEM

### B.1 Force equilibrium

The linearized equation for spatial force n (Equation 18) can be rewritten as,

(B1)
$$L[n]=\underset{C_{\mathrm{expn}}}{\underbrace{{\overset{*}{\Lambda }}_{t}C_{N}\overset{*}{\Gamma }}}+\underset{C_{\mathrm{nw}}}{\underbrace{\left({\overset{*}{\Lambda }}_{t}C_{N}{\left({\overset{*}{\Lambda }}_{t}\right)}^{T}\right)}}\Delta w^{\prime }+\underset{C_{n\psi }}{\underbrace{\left[{\overset{*}{\Lambda }}_{t}C_{N}{\left({\overset{*}{\Lambda }}_{t}\right)}^{T}\hat{{\left(\overset{*}{r}\right)}^{\prime }}-\hat{L[\overset{*}{n}]}\right]}}\Delta \psi .$$

For a cell C (Figure 3), the forces are balanced across the internal faces (w and e) of the cell and using appropriate discretization schemes, the cell face values and cell‐face derivatives are expressed in terms of the cell‐center values (Equations 25 and 27, respectively). The explicit term ($C_{\mathrm{expn}}$) and the coefficients of $\Delta w^{\prime }$ and Δψ in Equation (B1) are directly calculated at the cell‐faces using face values of the kinematic quantities $r_{f}^{\prime },{\left(\Lambda_{t}\right)}_{f}$ and the strain measures $\Gamma_{f},K_{f}$; therefore, they need not be expressed in terms of the neighboring cell‐centers. Thus, the discretized force equilibrium (Equation 23) can be expanded and written in matrix‐form as,

(B2)
$${\left[A_{W}\right]}_{n}\left[\begin{array}{c} {\left(\Delta w\right)}_{W}\\ {\left(\Delta \psi \right)}_{W} \end{array}\right]+{\left[A_{C}\right]}_{n}\left[\begin{array}{c} {\left(\Delta w\right)}_{C}\\ {\left(\Delta \psi \right)}_{C} \end{array}\right]+{\left[A_{E}\right]}_{n}\left[\begin{array}{c} {\left(\Delta w\right)}_{E}\\ {\left(\Delta \psi \right)}_{E} \end{array}\right]={\left(R^{w}\right)}_{C},$$

where the coefficient matrices ${\left[A_{W}\right]}_{n}$, ${\left[A_{C}\right]}_{n}$, and ${\left[A_{E}\right]}_{n}$ are 3×6 matrices containing the contributions of W, C, and E cell‐centers, respectively, are given by,

(B3)
$${\left[A_{W}\right]}_{n}=\left[\begin{array}{cc} \frac{1}{L_{w}}{\left(C_{\mathrm{nw}}\right)}_{w} & \;-\gamma_{w}{\left(C_{n\psi }\right)}_{w} \end{array}\right],$$

(B4)
$${\left[A_{C}\right]}_{n}=\left[\begin{array}{cc} -\frac{1}{L_{e}}{\left(C_{\mathrm{nw}}\right)}_{e}+\frac{1}{L_{w}}{\left(C_{\mathrm{nw}}\right)}_{w} & \;(1-\gamma_{w}){\left(C_{n\psi }\right)}_{w}-(1-\gamma_{e}){\left(C_{n\psi }\right)}_{e} \end{array}\right],$$

(B5)
$${\left[A_{E}\right]}_{n}=\left[\begin{array}{cc} \frac{1}{L_{e}}{\left(C_{\mathrm{nw}}\right)}_{e} & \;\gamma_{e}{\left(C_{n\psi }\right)}_{e} \end{array}\right].$$

The residual forces evaluated at the cell center C is given by,

(B6)
$${\left(R^{w}\right)}_{C}=\gamma_{w}{\left(C_{\mathrm{expn}}\right)}_{w}-\gamma_{e}{\left(C_{\mathrm{expn}}\right)}_{e}-f_{C}L_{C}.$$

### B.2 Moment equilibrium

The linearized spatial moment equation (19) and the linearized counterpart of the term $\left(r^{\prime }\times n\right)$ in Equation (20) can be expressed as,

(B7)
$$L[m]=\underset{C_{\mathrm{expm}}}{\underbrace{{\overset{*}{\Lambda }}_{t}C_{M}\overset{*}{K}}}+\underset{C_{m\psi }}{\underbrace{-\hat{L[\overset{*}{m}]}}}\;\Delta \psi +\underset{C_{m\psi 2}}{\underbrace{\left({\overset{*}{\Lambda }}_{t}C_{M}{\left({\overset{*}{\Lambda }}_{t}\right)}^{T}\right)}}\;\Delta \psi^{\prime },$$

(B8)
$$L[r^{\prime }\times n]=\underset{C_{\mathrm{expmn}}}{\underbrace{\hat{{\left(\overset{*}{r}\right)}^{\prime }}\;{\overset{*}{\Lambda }}_{t}C_{N}\overset{*}{\Gamma }}}+\underset{C_{\mathrm{mnw}}}{\underbrace{\left[\hat{{\left(\overset{*}{r}\right)}^{\prime }}\left({\overset{*}{\Lambda }}_{t}C_{N}{\left({\overset{*}{\Lambda }}_{t}\right)}^{T}\right)-\hat{L[\overset{*}{n}]}\right]}}\;\Delta w^{\prime }+\underset{C_{\mathrm{mn}\psi }}{\underbrace{\hat{{\left(\overset{*}{r}\right)}^{\prime }}[{\overset{*}{\Lambda }}_{t}C_{N}{\left({\overset{*}{\Lambda }}_{t}\right)}^{T}\hat{{\left(\overset{*}{r}\right)}^{\prime }}-\hat{L[\overset{*}{n}]}]}}\;\Delta \psi .$$

For a cell C, the moments are balanced across the internal faces w and e and, the discretized moment equilibrium equation (24) can be expanded as,

(B9)
$${\left[A_{W}\right]}_{m}\left[\begin{array}{c} {\left(\Delta w\right)}_{W}\\ {\left(\Delta \psi \right)}_{W} \end{array}\right]+{\left[A_{C}\right]}_{m}\left[\begin{array}{c} {\left(\Delta w\right)}_{C}\\ {\left(\Delta \psi \right)}_{C} \end{array}\right]+{\left[A_{E}\right]}_{m}\left[\begin{array}{c} {\left(\Delta w\right)}_{E}\\ {\left(\Delta \psi \right)}_{E} \end{array}\right]=\left[\begin{array}{c} {\left(R^{\psi }\right)}_{C} \end{array}\right].$$

The coefficient matrices ${\left[A_{W}\right]}_{m}$, ${\left[A_{C}\right]}_{m}$ and ${\left[A_{E}\right]}_{m}$ are 3×6 matrices given as,

(B10)
$${\left[A_{W}\right]}_{m}=\left[\begin{array}{cc} -\frac{1}{2}\frac{L_{C}}{L_{w}}{\left(C_{\mathrm{mnw}}\right)}_{w} & \;-\gamma_{w}{\left(C_{m\psi }\right)}_{w}+\frac{1}{L_{w}}{\left(C_{m\psi 2}\right)}_{w}+\frac{1}{2}L_{C}\gamma_{w}{\left(C_{\mathrm{mn}\psi }\right)}_{w} \end{array}\right],$$

(B11)
$${\left[A_{C}\right]}_{m}={\left[\begin{array}{c} \frac{1}{2}\frac{L_{C}}{L_{w}}{\left(C_{\mathrm{mnw}}\right)}_{w}-\frac{1}{2}\frac{L_{C}}{L_{e}}{\left(C_{\mathrm{mnw}}\right)}_{e}\\ (1-\gamma_{e}){\left(C_{m\psi }\right)}_{e}-(1-\gamma_{w}){\left(C_{m\psi }\right)}_{w}\\ -\frac{1}{L_{e}}{\left(C_{m\psi 2}\right)}_{e}-\frac{1}{L_{w}}{\left(C_{m\psi 2}\right)}_{w}\\ +\frac{1}{2}L_{C}(1-\gamma_{e}){\left(C_{\mathrm{mn}\psi }\right)}_{e}+\frac{1}{2}L_{C}(1-\gamma_{w}){\left(C_{\mathrm{mn}\psi }\right)}_{w} \end{array}\right]}^{T},$$

(B12)
$${\left[A_{E}\right]}_{m}=\left[\begin{array}{cc} \frac{1}{2}\frac{L_{C}}{L_{e}}{\left(C_{\mathrm{mnw}}\right)}_{e} & \;\gamma_{e}{\left(C_{m\psi }\right)}_{e}+\frac{1}{L_{e}}{\left(C_{m\psi 2}\right)}_{e}+\frac{1}{2}L_{C}\gamma_{e}{\left(C_{\mathrm{mn}\psi }\right)}_{e} \end{array}\right].$$

The residual moment evaluated at the cell center C is given by,

(B13)
$${\left(R^{\psi }\right)}_{C}={\left(C_{\mathrm{expm}}\right)}_{w}-{\left(C_{\mathrm{expm}}\right)}_{e}-\frac{1}{2}L_{C}{\left(C_{\mathrm{expmn}}\right)}_{w}-\frac{1}{2}L_{C}{\left(C_{\mathrm{expmn}}\right)}_{e}-t_{C}L_{C}.$$

The diagonal and off‐diagonal coefficient matrix contributions from the force and moment equilibrium equations can be assembled and the coupled block linear system (Equation 29) coefficient matrices take the form as follows,

(B14)
$$A_{W}=\left[\begin{array}{c} {\left[A_{W}\right]}_{n}\\ {\left[A_{W}\right]}_{m} \end{array}\right];\;A_{C}=\left[\begin{array}{c} {\left[A_{C}\right]}_{n}\\ {\left[A_{C}\right]}_{m} \end{array}\right];\;A_{E}=\left[\begin{array}{c} {\left[A_{E}\right]}_{n}\\ {\left[A_{E}\right]}_{m} \end{array}\right].$$
